# The relationship between the mental health status and social support of the lonely elderly with government participation in the Internet context

**DOI:** 10.3389/fpubh.2022.1013069

**Published:** 2022-10-28

**Authors:** Jun Guo, Wenhao Ling

**Affiliations:** ^1^Department of Labor and Social Security, School of Philosophy and Public Administration, Henan University, Kaifeng, Henan, China; ^2^Institute of Social Governance Research Center, Henan University, Kaifeng, China

**Keywords:** mental health status, social support, the lonely elderly, government participation, Internet context

## Abstract

**Objective:**

At present, urban community aging has become a new way to solve aging problem and has made outstanding contributions to alleviate current aging dilemma. However, there are still deficiencies in the services provided by this new method of community-based elderly care for the elderly who are left alone. Therefore, this paper explores the problems of government policies, facilities and services for the elderly who have lost their independence, analyzes the reasons behind them, and proposes countermeasures.

**Method:**

This paper introduces and compares the government's current services for the elderly who are left alone in the city at the level of community-based elderly care services. From the existing literature, we find that most researchers have studied the single-parent family as an individual, exploring its elderly care dilemma, analyzing the causes and proposing countermeasures. It introduces and summarizes the situation of the elderly left alone in Taiyuan, the government's policies on the elderly left alone and community elderly care, the current community elderly care construction in Taiyuan, and the service models provided by the government.

**Results/Discussion:**

This paper provides a detailed description and in-depth analysis of the lack of elderly care protection in communities, and analyzes the underlying causes. Activity theory suggests that even though older people are no longer in the same mental state as younger people, they can still enhance their self-worth through active participation in social activities and gain a sense of social identity through social activities. The services provided by social organizations are typical services purchased by the Health and Welfare Commission for the elderly who are left alone, and it is feasible to gradually extend the services to provide more specialized community-based elderly care services for the elderly who are left alone. Therefore, this study takes the use of services provided by social organizations as an example to understand the current situation of services received and used by the elderly who are left alone, and concludes that the services are weakly accessible, and finally proposes suggestions to improve the accessibility of services in three aspects: supply, delivery, and use.

## Introduction

In a theoretical sense, as the aging of China becomes more and more severe, the number of one-child families is gradually increasing and has become the main family structure in urban areas (1). Secondly, in the practical sense, if it is not possible to effectively solve the many problems of elderly care for the lost-alone families, it will not only affect the quality of life and happiness index of the lost-alone families, but even hinder the social harmony and stable development (2). Moreover, the lost-alone families have something in common with the elderly groups such as living alone and empty nesters, and providing good elderly care protection for lost-alone families also provides new inspiration for other elderly groups (3). The study of community elderly care service is not only beneficial to integrate the existing elderly care resources and relieve the pressure of elderly care, but also beneficial to the state to better provide elderly care services for the elderly who are left alone, and effectively solve the problems of living, economic, spiritual, medical and elderly care for the families left alone (4).

By 2030, an estimated 851,000 parents who have lost a single child will be found in rural areas and 572,000 in urban areas (5). As they age, the issue of living and caring for the elderly in these families becomes an increasingly important issue (6). Social policies have provided some financial subsidies for the elderly who have lost their only child appropriately, but the elderly who have lost their only child still suffer from broken support networks, lack of support from their children, lack of support from neighbors and relatives and friends; elderly care services suffer from poor linkage and continuity, low recognition by social workers, and slow in carrying out their work (7). The government and academia have discussed and explored the issue, and agreed that home care is the most suitable way for the elderly in China and the traditional thinking. According to the Law on the Protection of the Rights and Interests of the Elderly introduced in 2007, “The state shall establish and improve a system of elderly care services based on families, streets and institutions.” However, since social work intervention in elderly care services for the lost elderly is at a preliminary stage, it is unable to address the diverse needs of the lost elderly in practice, and there are also problems such as insufficient service provision and low professionalism and accuracy of services (8). Therefore, how to improve the overall level of social work intervention in elderly care services for the lost elderly is the main issue at present. We should not only see the risks and problems of the elderly who are left alone, but also pay attention to the expression of needs and capacity building of the elderly who are left alone (9). This dissertation focuses on mutual help support for the elderly who have lost their independence (10). The reason for adopting this research point is that as the number of the elderly who have lost their children continues to increase, the community has begun to care for the elderly who have lost their children in various ways, and also realize that the loss of their children is a fatal blow to them, but the elderly who have lost their children are also independent individuals who have advantages in many aspects, and the different advantages can be aggregated together for mutual support (11).

The support for the elderly who are left alone requires a certain opportunity to bring this special group together and a professional approach and means to solve the material and spiritual problems of the elderly who are left alone. In my practice, we have found that social work can provide services for the elderly who have lost their independence from a professional perspective, combine the local cultural background with the problem of lost independence, link policy resources, and link multiple departments, and use professional social methods to design a series of hierarchical and targeted activities for the elderly who have lost their independence. Based on the previous research, this paper takes the elderly alone as the main subject of the study. The social workers integrate the elderly alone into a mutual help group, and use professional working methods and resources to transform the elderly alone from a passive recipient of assistance to an active participant in mutual help and support, effectively reducing the grievances of the elderly alone toward the government, helping them to relieve their emotions, gradually get rid of the shadow of the elderly alone, and reintegrate into society. In the process of reading the literature and participating in the practice, this paper considers that the services provided by the government, the community and public welfare organizations for the elderly who have lost their independence are very sensitive and fragile due to the breakdown of family structure, and it is difficult to achieve the desired effect. This paper takes social work theory as the starting point to observe the interaction between the elderly alone and the social work agencies, and investigates in depth the process of social work interventions in mutual support for the elderly alone.

## Related work

First of all, in the early legal documents, the term “one-child family with disability and death” was used, which in essence defines the concept of “lost family,” i.e., a family that is a one-child family due to the family planning policy implemented (12). This is a family that has not had another child or adopted a child after the death of the only child (13). The definition of the single elderly person in legal documents has become a commonly accepted concept among scholars, and it is also the main criterion for the government to define the protection of the single elderly person. Secondly, researchers in the academic field have used different terms for single-parent families, including “single-child families,” “single-parent families,” “single-parent elderly,” and “single-parent families,” etc., and have conducted research on related topics. Even though different researchers have used different terms, there is no debate about the substantive connotation of the single-parent family. Scholars have categorized the concept of lost-alone families from the perspective of conceptual categories, such as Professor Mu Guangzong's classification of lost-alone families into three types of families under three criteria: firstly, the first category is divided into “absolute lost-alone families” and “relative lost-alone families” based on whether the only child has died or not The first category is based on whether or not the child died, and is divided into “absolute families” and “relative families” (14). The second category is divided into “de facto” and “potential” families based on the occurrence of the “loss of independence” event (15).

A “de facto family” refers to a family in which the only child has died, while a “potential family” refers to a family in which the child is still alive but may be at risk in the future. The third division is based on whether the one-child family is related to the family planning policy, and the one-child family is a “policy family” because it follows the national policy of having only one child (16). In this paper, the elderly who have lost their own children are referred to as “policy-lost families.” In this paper, the group of elderly people without a child is defined as a family that has only one child or adopted a child under the family planning policy, but after the death of the child due to the main or objective reasons, the family does not have another child or adopt another child due to various reasons, which is a “family without a child” in the narrow sense (17). The concept of community-based care is based on the concept of “community care” first introduced in the UK. The concept of “community care” was first introduced in the United Kingdom, but the introduction of the community platform on the basis of family elderly care has developed into a service model that meets the actual needs of the elderly (18).

It was first proposed in the published “Building a Community Aging-in-Home System,” which has attracted extensive focus from scholars after it was first proposed, while scholars have discussed in depth the specific role of the community in the elderly care (19). In the process of providing elderly services, the role of the community is proposed to integrate the various resources in the services, and the elderly care model is based on family aging and assisted by the community. Through further in-depth discussion, it is proposed that the focus of community-based elderly care services is the intermediary role of the community (20). In other words, the community is responsible for the integration and transfer of government or social resources for the elderly; at the same time, as one of the main service providers, the community is responsible for meeting the needs of the elderly in the community. Therefore, “community” is not only a platform to connect government, society and the elderly, but also a main body to provide services to the elderly, presenting a double function.

## Government-led community-based elderly care services for the elderly left behind in urban areas

### Community aging service model

**Figure 1:**
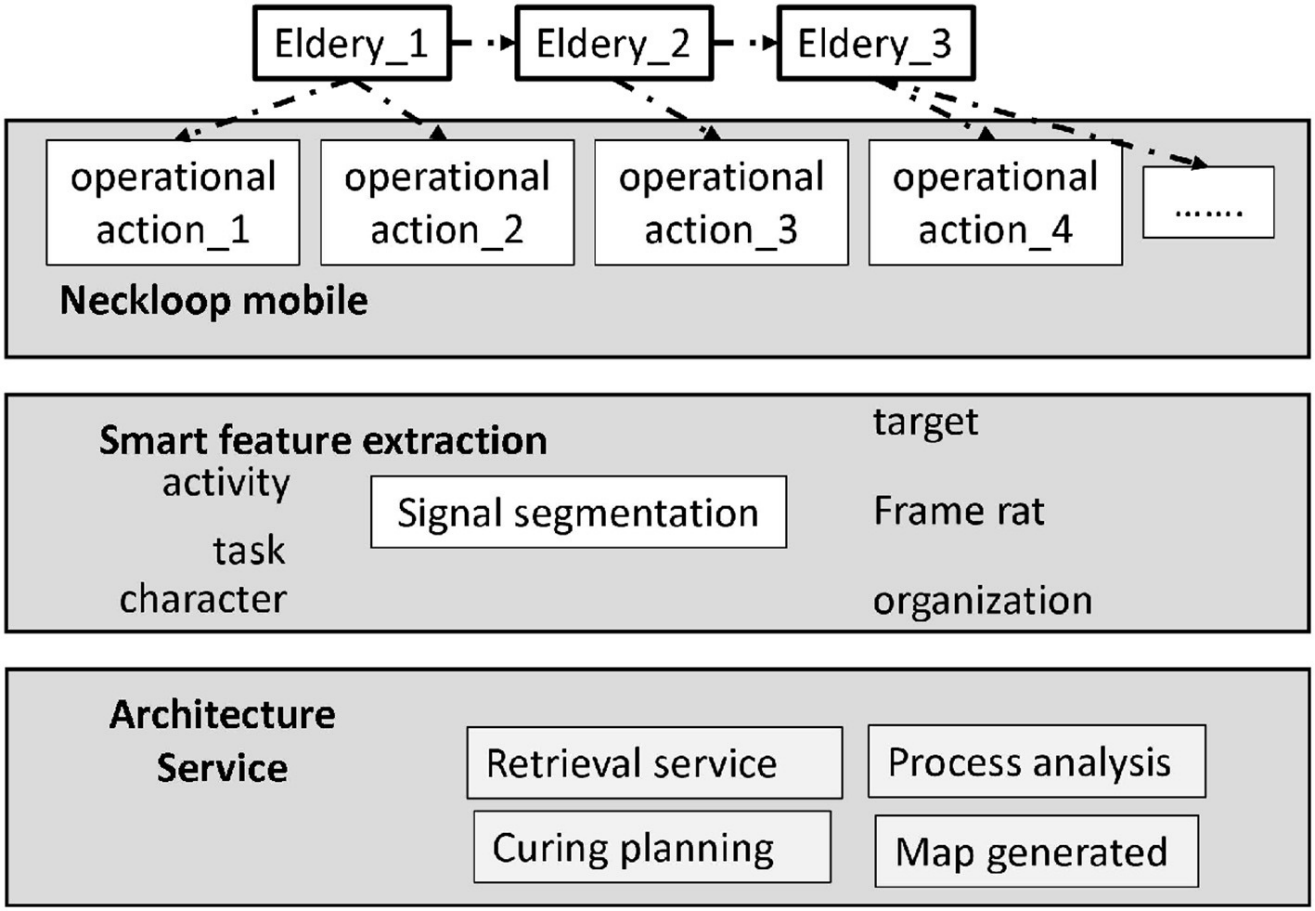
Community-based elderly care service model for the elderly who have lost their lives.

Community senior care service is mainly based on the family as the core, the community as the platform, through the community as a platform to integrate multiple resources, and strive to fully meet the needs of the elderly in the community. So far, there are corporate, institutional and pure residential community senior care service models. Among them, the corporate type and the institutional type have a higher degree of service perfection, while the number of pure residential type accounts for the most. Each of the three types of community senior living models has its own characteristics. First of all, the main service group of enterprise-type community elderly care is the retired employees of state-owned enterprises. The Taiyuan Iron and Steel Group is more prominent in securing senior care services. They have set up a special activity room for the elderly in the community and a health service center to provide exclusive catering, medical care and recreational activities for the elderly in the community. Secondly, the main group of people served by the organ-based community pension is the retired old cadres in Taiyuan. The “one-touch call” model is built in the community, and a one-touch “electronic babysitting” system is installed for the elderly for free. By building this system, the elderly in the community can be provided with meals, medical and health services at any time, as shown in Figure 1.

Approved by the Hospital of Medical College, this study selected patients who underwent general anesthesia at the Department of Oral and Maxillofacial Surgery from June 2021 to December 2022 and had three or more impacted oral health extracted at one time. There are 90 patients in total, aged 18–25 years, 45 males and 45 females. All patients were in good health before admission, and the contraindications of tooth extraction and general anesthesia were excluded.

Lastly, the number of purely residential communities is the largest of the three types, where services are provided to the general population of 60-year-olds. In order to provide better services, the government has launched pilot projects in the Riffen Yuan community, the Old Military Camp 3 community, the Taobei East community, and the Binhe community. The purely residential type has set up a community service network platform in the community and refined the service targets according to the degree of difficulty, including those who are over 80 years old; those who are financially disadvantaged and receive the national low income insurance; and those who are over 60 years old but do not have financial difficulties. For these three categories of service recipients, the community will provide targeted compensation services. The first and second categories of elderly people can enjoy government subsidies of 100 yuan and 50 yuan, respectively; the third category of elderly people can experience the government's affectionate elderly services once, including services such as hygiene cleaning, legal assistance and goods distribution and maintenance. After the experience, the elderly can continue to choose the paid services by making appointments by phone if they need. This study introduces the concept of “accessibility” to the field of community elderly care services. Based on the existing accessibility analysis model, the analysis framework of this paper was built, and the interview outline was designed based on this framework to obtain first-hand data on the perception of accessibility of the elderly left alone.

This study is based on the project of “Care for the elderly who have lost their only child”, and explores the process and content of the activities of the oral health for the elderly who have lost their only child, the path of building a mutual support network for the elderly who have lost their only child. In order to make it more convenient for the elderly to use and to enrich the service content, the community elderly service card is issued, and the elderly with the card will enjoy the “six help” services of emergency assistance, medical assistance, meal assistance, cleaning assistance, bath assistance and shopping assistance (walking). First of all, the elderly can propose service needs to the senior service hotline with the card, and the hotline will dispatch corresponding services according to the needs proposed by the elderly. After enjoying the services, the elderly can use the service card to pay. Secondly, the scope of services provided by the card is divided into two types of services: basic senior care services and basic living services. The basic elderly services include emergency assistance and medical assistance; the basic living services include meal assistance, cleaning assistance, bathing assistance and shopping assistance (walking). Finally, the government purchases senior care services from the society to provide senior care services for the elderly in the community and play a bottom-up role. The Taiyuan community provides a monthly service subsidy of 100 RMB per person for the elderly in the community. 40 RMB of the 100 RMB can be used for basic senior care services such as emergency assistance and medical assistance; the remaining 60 RMB can be used for basic living services of the elderly's own choice. In other words, the community is responsible for the integration and transfer of government or social resources for the elderly; at the same time, as one of the main service providers, the community is responsible for meeting the needs of the elderly in the community. Therefore, “community” is not only a platform to connect government, society and the elderly, but also a main body to provide services to the elderly, presenting a double function.

Seniors can choose their own according to their actual needs and pay for the excess by themselves. The project of “one park and two centers” has been built. First of all, the “one park” refers to the incubation park of the community elderly service industry chain, which was funded by the government. The project provides one-stop services for community elderly care through the government, such as free training, legal and policy consulting services, etc. At the same time, a special zone is set up to provide project guidance, encourage enterprise innovation, and gradually introduce international and domestic branded and chain service enterprises to form a community elderly care service industry incubation park. Secondly, the “two centers” refer to the Binhe Fruitland Community Senior Service Center and Wulongwan Day Care Center invested by Taiyuan Municipal Government. The focus of the “two centers” is to provide diversified elderly service needs to the community, establish service institutions that meet the needs of the elderly, and set a benchmark for the construction of community elderly service institutions. In addition to the above functions, the project will also become a standardized demonstration training center for community elderly care in Taiyuan. Finally, Taiyuan City introduces foreign professional service organizations to the city to establish a municipal intelligent community senior care service platform to carry out home meal preparation, psychological counseling, physical examination and other senior care services; to provide professional and quality services for the elderly in the city, such as into Shanghai Easy Care, Nanjing Sankyo Group, Shandong Qingdao, etc. The government provides protection for the elderly with financial difficulties by purchasing community elderly services, and relies on community elderly service centers and day care centers to supply more than 23,000 elderly with six types of services, including emergency assistance, medical assistance, meal assistance, cleaning assistance, bathing assistance and (shopping) assistance, with the number of service visits exceeding 2 million.

### Orderly construction of mutual support network

**Figure 2:**
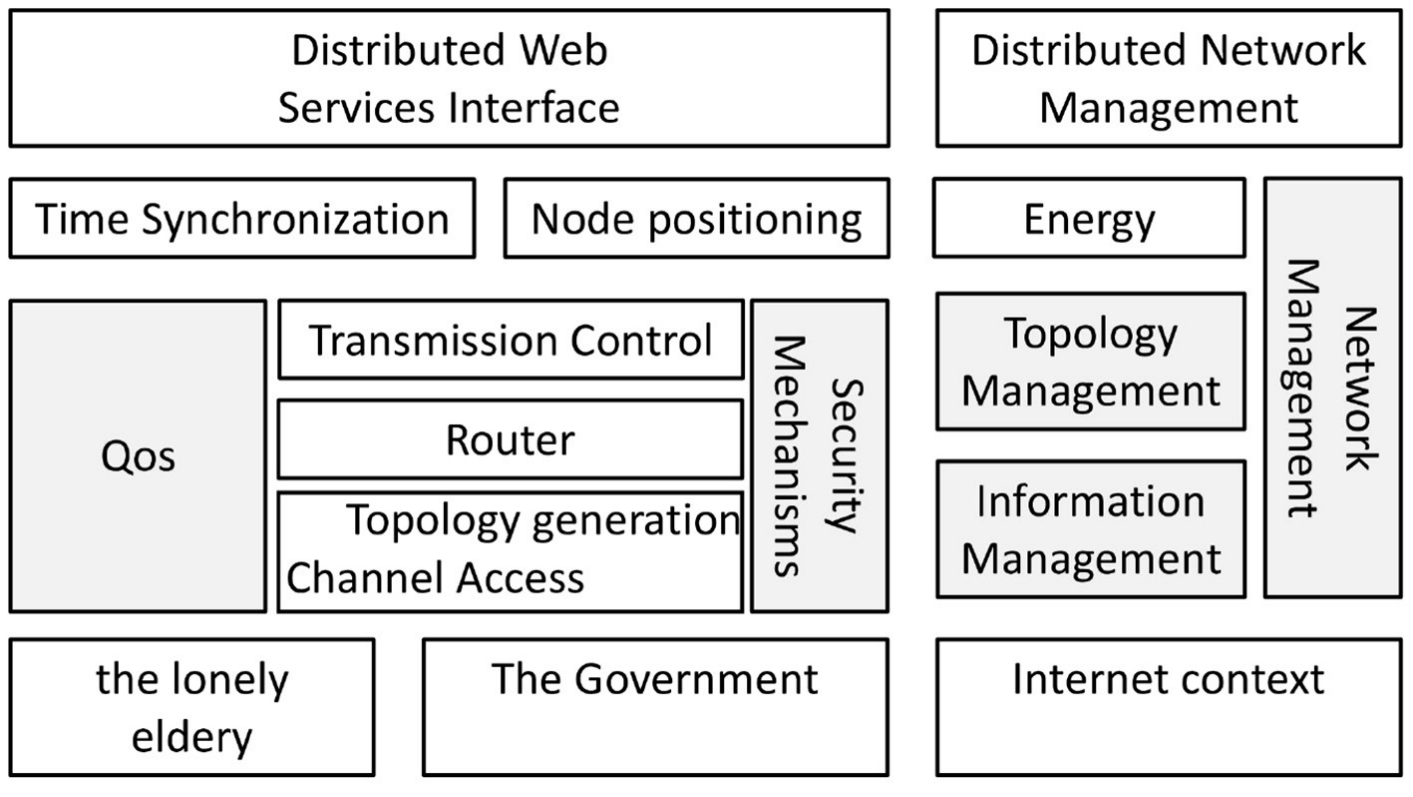
Action strategy framework.

This paper uses social capital theory as a guide, and applies the three dimensions of trust, network, and norms to the process of building a mutual support network for the elderly who have lost their lives. At the same time, social work, as a professional third-party interventionist, uses the value of self-help guidance and professional and specific work methods to help the elderly who have lost their lives to overcome their grief, strengthen mutual support, and actively integrate into society, as shown in Figure 2. In other words, we assess the needs of the elderly who have lost their lives based on their current situation, and based on their needs, we help them to face their lives again and work together to help more “people with the same fate.” This chapter introduces the practical strategies for the orderly construction of a mutual support network, mainly focusing on the following questions: Why choose social work intervention? What methods do social workers adopt to gain the trust of the elderly who have lost their lives? What activities do social workers design to help them build a mutual support platform? How to make the mutual support network for the elderly alone sustainable? In this paper, we combine the elderly who have lost their independence with community-based elderly care, and explore the top-level design. In the process of community-based elderly care construction, the government can better guarantee the elderly care for the single-parent families.

Formal support can provide financial, environmental and life security for the elderly; informal support can make good use of the social network resources of the elderly and comfort them in oral health. As older adults gradually withdraw from the social stage as they age, they change from being participants in social activities to being bystanders, and they lack support.

Before the intervention of social workers, the government mainly “covered” the problem of the elderly who were left alone by formulating macro policies, and also turned to civil affairs, community family planning and women's federations. There are many elderly people who have moved away from their original communities and their homes are scattered, so it is difficult to centralize services. For those who need special assistance, or need to accompany the elderly to and from the hospital for major illnesses, the community currently lacks staff and funds, making it difficult to implement. Some of the old people who have lost their independence are self-absorbed, seldom go out, refuse the intervention of others, and their quality of life is declining. It is believed that the elderly who are left alone are now managed and served by the family planning department, while other departments have no responsibilities and are busy, and there is no specific method of multi-sectoral cooperation. It is believed that multi-sectoral cooperation is necessary, but it needs to be supported by the establishment of policies and responsibilities. Social work agencies, however, can provide professional and diversified services as a medium to link up multiple agencies, using their professional skills and working methods to design activities and link up multiple resources according to the multi-level needs of the elderly.

**Figure 3:**
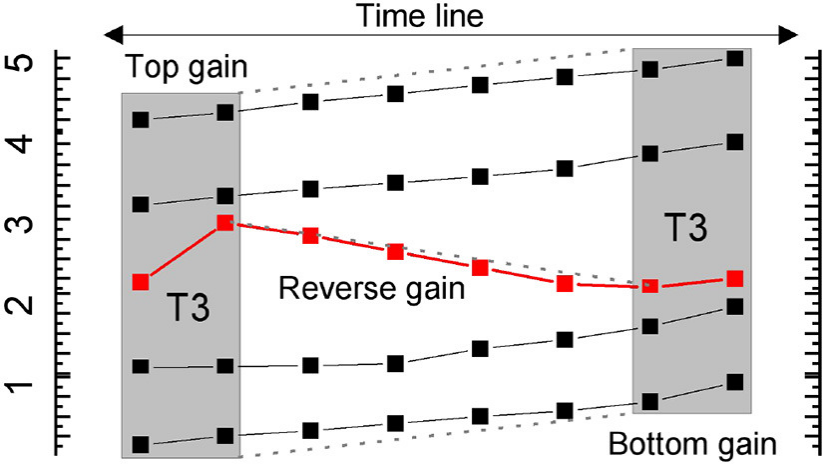
Social organization project contact person's license status table.

At present, the contact persons of social organizations are mainly the retired residents of the streets, which can account for 60% of the total number of contact persons. Although the retired cadres play a very important role in the actual work, compared with other contact persons, the retired cadres understand the actual situation of the streets and can deal with the trivial matters in the streets, however, in terms of professional theoretical knowledge, the contact person team in the streets still needs to be further strengthened, and the current social work certificate holding rate is low, and the number of licensed social workers in each street is shown in Figure 3.

### Social adaptation of the elderly living alone in the context of the Internet

**Figure 4:**
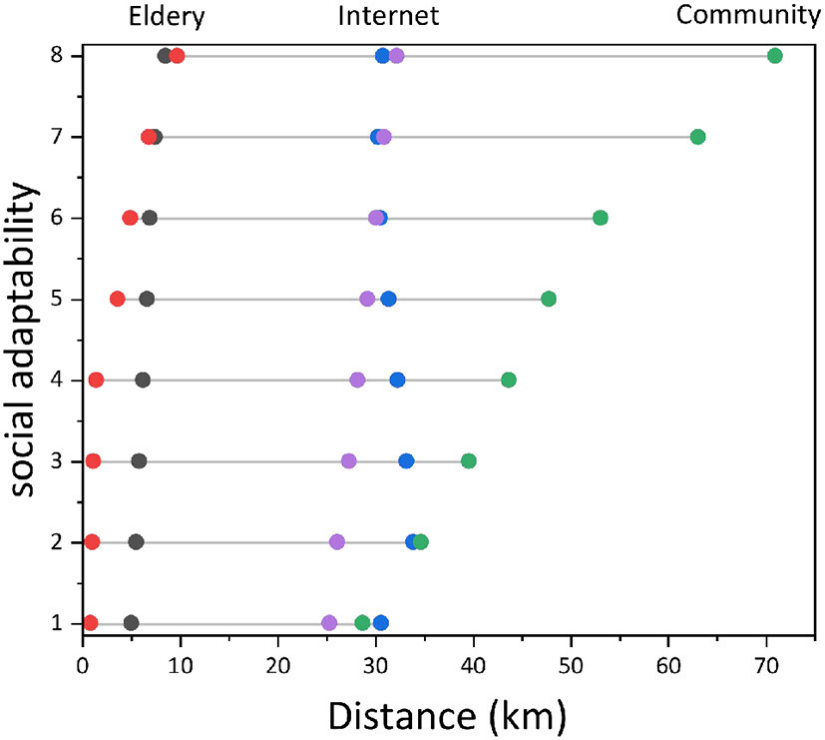
Summary analysis results by category.

Through questionnaires and interviews, it was found that there is a group of people in community X who live alone in the community without children or partners, and who are not only emotionally unavailable, but also have a gap in adaptation with their peers in the context of the Internet society. This study focuses its research on the population of elderly people living alone. At the same time, I also found that the problems brought by elderly people living alone are more obvious and difficult to cope with than those accompanied by family members. As shown in Figure 4, it is a survey on the social adaptability of the elderly in the context of the Internet. 40 valid samples were collected, and the content of the survey was studied in terms of the age of the elderly, the way of living, the use of smartphone channels, online shopping, and travel sweeps. If we divide the survey according to the level, 2.25–3.0 is qualified, 3.0–3.75 is good, and more than 3.75 is excellent. The level of social adaptability of the elderly on the Internet is all pass, and all of them reach excellent in mobile payment, but all of them are average in other aspects, especially compared with the elderly with children and with partners, the elderly living alone are lagging behind in using online shopping, traveling with a code, and paying bills online. As for the needs of the elderly living alone, some of them hope to make new friends through the Internet and learn to communicate with their children through Internet video; in terms of information acquisition, some elderly living alone hope to get international and domestic development trends from the Internet and pay attention to major national events.

**Figure 5:**
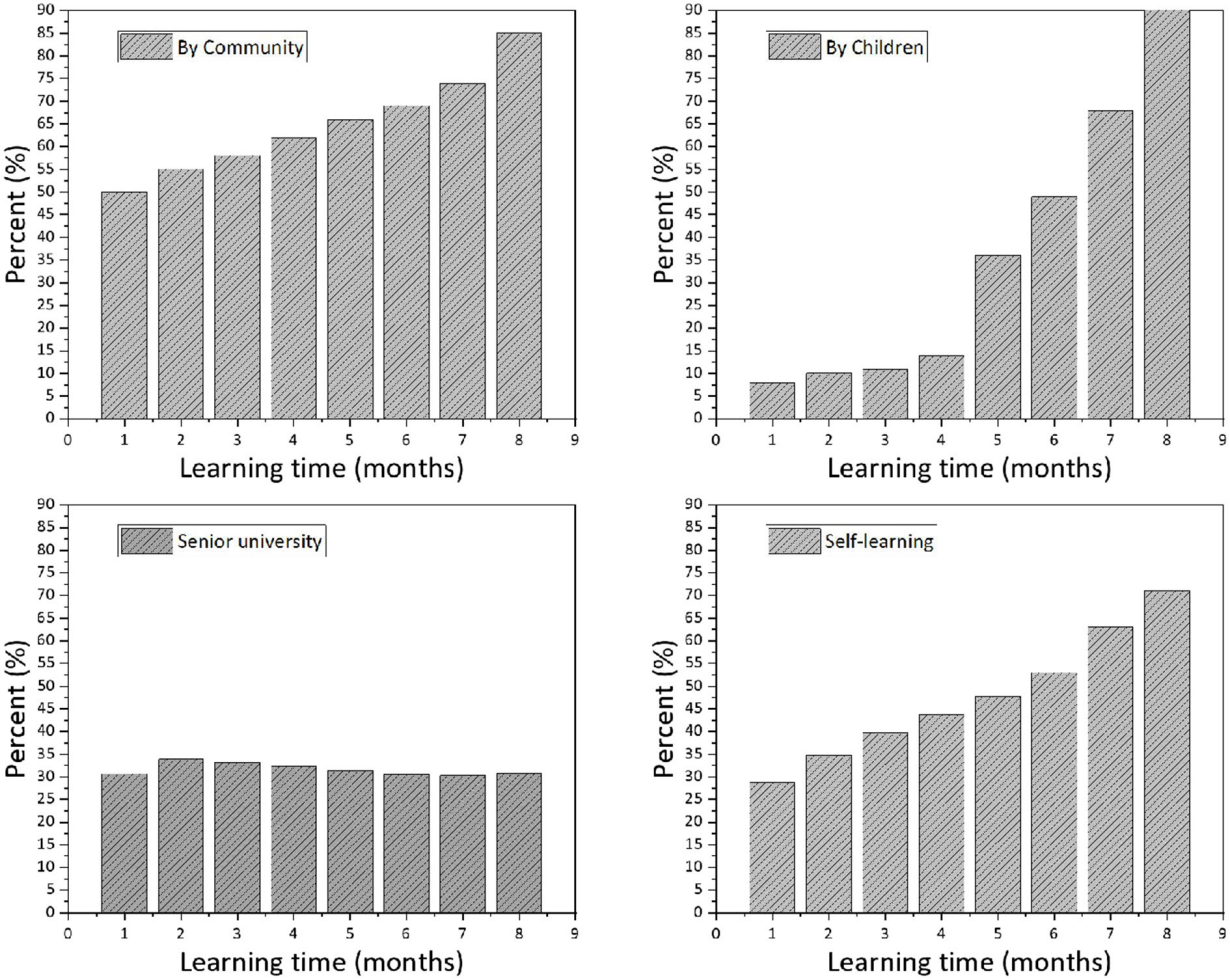
Smartphone learning channels for older adults.

Nowadays, everyone cannot live without the Internet and the use of smartphones to adapt to the Internet society, and the elderly are no exception. According to the questionnaire survey, the adaptation of the elderly living alone in the context of the Internet, compared with the elderly living with their children or with their partners, the elderly living alone are generally adapted to the use of online shopping and social software, less adapted to booking and social. In terms of booking appointments and social entertainment, they are not so comfortable with it, and they are very uncomfortable with going out for online appointments. The author learned from the interviews that most of the elderly living alone are at the primary stage of using internet tools, and due to the lack of personnel to teach them how to use them systematically, they have only heard about the functions of daily convenience and social affairs. On the whole, elderly people learn to use smartphones mainly through self-learning, followed by children's teaching, community teaching, and senior college. As for the use of smartphone channels by the elderly living alone, self-learning is the main channel for the elderly, followed by teaching by relatives and friends, and only 6.25% of the elderly choose the community service center, which shows that the current lack of Internet teaching environment for the elderly, only by self-learning, it is difficult to fully understand the use of network tools and adapt to the Internet society. As shown in Figure 5, the chi-square test (cross-tabulation analysis) shows that the residence mode does not show significance (*p* > 0.05) for senior college, which means that the different residence mode samples show consistency and no difference for learning through senior college. The residence mode sample showed significance (*p* < 0.05) for self-study, children's teaching, and community teaching, meaning that the different residence mode samples showed differences for self-study, children's teaching, and community teaching, and the residence mode showed 0.05 level of significance for self-study (chi = 8.051, *p* = 0.018 < 0.05). Through several months of internship and contact with the elderly, the author found that some of the elderly living alone, in the context of the Internet society, have poor social adaptability, and their learning conditions are not as good as those of the elderly with family members, both in terms of travel and in terms of life.

**Figure 6:**
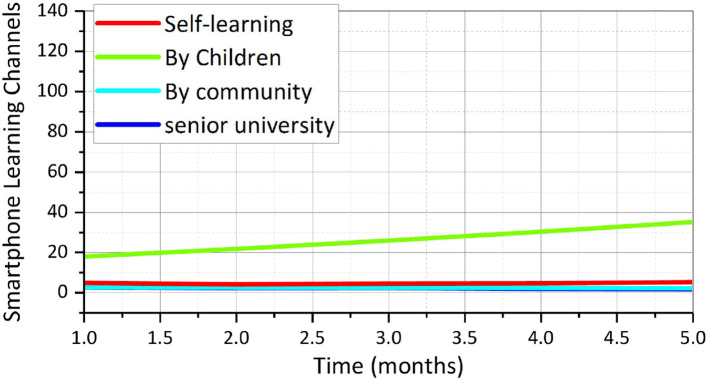
Smartphone learning channels for older adults with no residence patterns.

In terms of learning needs, most of them show a strong interest in learning and hope to be able to travel without the help of others and do it independently by themselves. They also want to make good use of the Internet to get more health tips, and a few of them are improving their quality through learning. It can be concluded that most of the elderly living alone in community X are eager to be exposed to the Internet society, to improve their personal problem-solving skills, and to achieve better social adaptation. However, due to the lack of educational environment, the use of Internet tools by the elderly living alone is in its infancy, and they do not fully enjoy the convenience brought by technology to their lives, but have certain social adaptation problems in the rapidly developing Internet society. The author conducted an in-depth study of 10 elderly people living alone, as shown in Figure 6, and through interviews with them, gained a deeper understanding of the group's ability to adapt and adapt to the situation. Among the 10 service users, 30% of them were male and 70% were female, and most of them had education level below junior high school, and the questionnaire survey showed that although the education level was low, all of them knew Chinese characters and most of them were keen on handwriting.

In terms of social interaction, most elderly people who live alone in their old age stay at home and have little contact with friends in real life and almost no contact in the Internet. Therefore, it can be concluded that some elderly people living alone have difficulties in social interaction. In terms of daily life: the use of Internet tools by the elderly living alone is not very good. Some basic functions, such as travel codes, are difficult for the elderly living alone, which makes it more difficult for them to travel. In terms of Internet exposure: Some of the elderly living alone are resistant to the Internet, believing that they are too old to live alone and do not need to integrate into the Internet society. This reluctance to accept new things and even self-denial hinders the integration of the elderly living alone into the Internet society. After analyzing the survey results, the author summarizes the problems of social adaptation of the elderly living alone in X community in the context of the Internet society.

## Discussion

**Figure 7:**
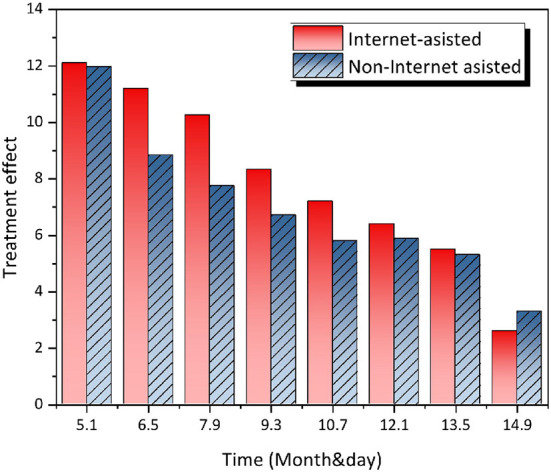
Internet social participation of the elderly living alone.

According to the actual survey, the social communication of the elderly living alone in community X of S city is superficial and the frequency of social activities is low. Social participation is very rare. In the research, I found that the activities held by the activity center in the community never involved activities to help the elderly adapt to the Internet society, and the activity center was often closed, and the staff said that it was only opened during specific activities, but not during normal times, and most of the activities had fixed members. For the elderly living alone, it is necessary to go out and meet new people to enrich their physical, mental and leisure life. A questionnaire survey was conducted on the social participation of 10 elderly people living alone, and the details are shown in Figure 7. The survey on the social adaptation of the elderly living alone showed that the elderly living alone in community X had different indicators of social life on the Internet, which were strongly agree, moderately agree, average, not very agree, and strongly disagree. The elderly living alone generally believe that the Internet society has a great impact on their lives, and it involves a wide range of aspects, such as travel, friendship, children's communication, and social and political affairs. Only 10% of the elderly living alone are proficient in the use of smart phones, 90% of the elderly living alone are not proficient in the use of smart phones, including the need for young people to help scan the code, sick registration children are not around to register in advance in the cell phone registration can only queue to the hospital registration, in addition, for the elderly living alone using cell phone dating is also a problem, the elderly can not add friends This means that it is difficult for the elderly living alone to adapt to the Internet environment.

**Figure 8:**
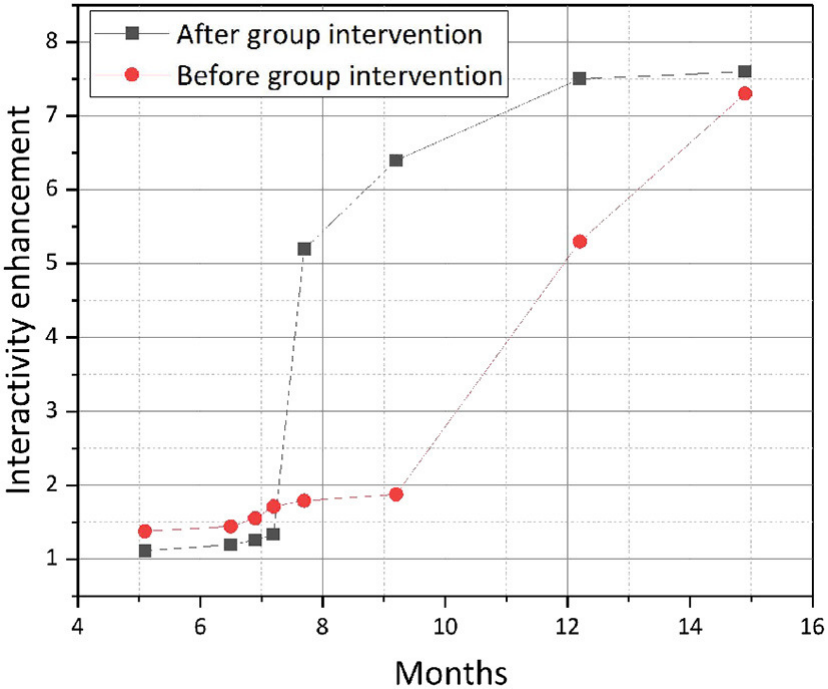
Basic indicators of adaptation to the network environment before and after the group's intervention.

Learning to use the Internet can facilitate the lives of seniors living alone and make their leisure time more exciting. Not only can they learn new knowledge and understand various policies, but they can also shop and invest. However, if they do not learn to use the Internet, they will not get the dividends brought by a smart society and will bring more inconvenient life effects to future generations. Since the elderly children are not around, the elderly living alone lack the learning conditions taught by their family members, bringing more inconvenience. For example, what is such a simple and easy operation for us to travel by sweeping a pass code is very difficult for some elderly people living alone, which makes them have to ask staff for advice or make temporary registration. The Internet may reinforce negative stereotypes of the elderly, who are not as comfortable using the Internet as younger people, are often overwhelmed when they encounter problems, often need to ask for help, and are inefficient in solving problems independently. More seriously, it may even affect some elderly living alone to deny their self-worth, thus affecting the quality of life of the elderly. As shown in Figure 8, in terms of adapting to the Internet environment, this study listed four dimensions, the ability to adapt to the Internet society, the impact of smartphones on life, the impact of the population on policies, and the impact on social aspects, the mean value on each dimension increased and was large. Thus, overall, the group members' ability to adapt to the Internet society has improved, and they are able to actively participate in social activities, which is a significant improvement.

**Figure 9:**
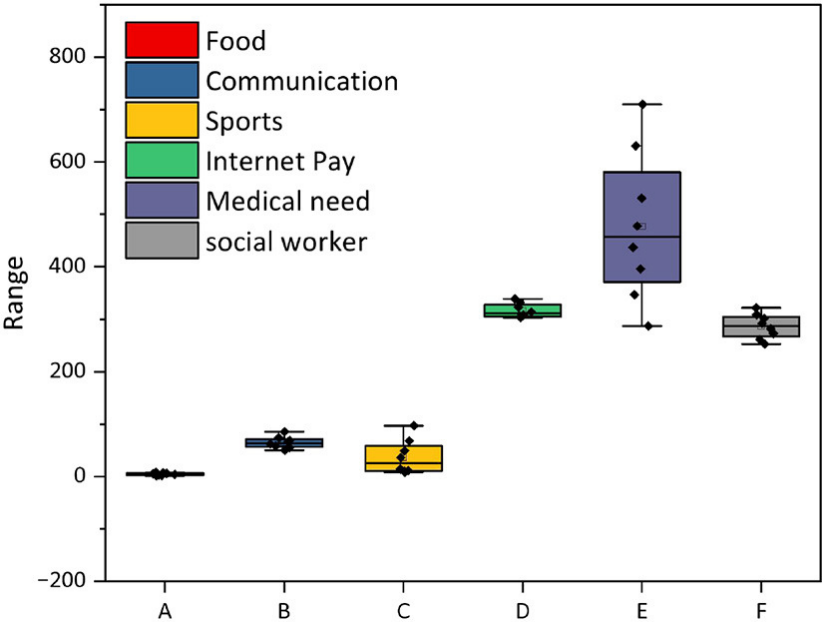
Baseline indicators of efficiency in addressing adaptation issues before and after government intervention.

Figure 9 below shows that each of the indicators of adaptive problem solving efficiency, i.e., the ability to behave in the Internet society, improved after the study group intervention, with a median score of 4.00. This means that the study group intervention had a good effect on the problem solving efficiency of the elderly living alone. Before the group intervention, most of the group members had to ask for help in getting around, but after the group intervention, according to the data, the group members were able to master the ways and means of getting around, and the effect was obvious. It is clear that the stress coping level of the elderly living alone has steadily increased after the group intervention, with the average increase ranging from 0.7 to 1.4, with the greatest increase in the problem solving dimension, which indicates that the elderly living alone have a great change in their problem solving attitude and are able to solve problems independently within their own ability. On the whole, the social adjustment index of the elderly living alone in the Internet context improved after the group work intervention, i.e., the social adjustment ability of the elderly living alone in the Internet improved after the group work intervention. Specifically, the effect of improving the subjective well-being of the elderly living alone was not obvious; the elderly living alone were interested in new things for a while, but the effect was not obvious in the long term.

## Conclusion

In the broader context, the lost-alone family is a result of China's family planning policy, and the issues related to this group require our practical attention from their perspective. The core problem of the elderly who have lost their only child is a series of problems and difficulties caused by the loss of their only child, and we need to think from the core when we consider solving their difficulties. There are still some shortcomings in the community-based elderly care, which is a social problem for the elderly who have lost their only child. Although Taiyuan City is at the forefront of Shanxi Province in the provision of community-based elderly care services, which have alleviated the plight of some elderly people in their old age, there are still shortcomings for the elderly who are left alone. This paper addresses this situation by sorting out and analyzing the shortcomings that exist within community-based elderly care for the elderly who are left alone from the government's perspective, while proposing solutions to the problems that have existed for the elderly who are left alone in Taiyuan City in the community, in the hope of helping the elderly who are left alone to solve their elderly care dilemmas, improve the quality of elderly care services, and enjoy their old age without fear. The purpose of this study is to improve the social adaptation ability of the elderly living alone in the context of the Internet, hoping that the group members can master the skills of solving problems in the Internet, change their mentality, and be able to improve both the internal experience and external performance of psychological adaptation. Looking back at the whole group activity, as a social worker, we should master the following skills. Firstly, in the operation part, the order of learning should be reasonable to ensure that each group member can have a sense of participation and consider the feelings of each member. Secondly, in the setting of the activity content, the design of each activity link should be reasonable and logical, the articulation of activities should be reasonable, the difficulty should not be too high, easy for the group members to master, and can stimulate the self-confidence of the group members, the activity design should be gradual, and in the process of setting rewards, attention should be paid to the emotions of all group members to ensure the effectiveness of the activity. In the future, everyone cannot live without the Internet and the use of smartphones to adapt to the Internet society, and the elderly are no exception. According to the questionnaire survey, the adaptation of the elderly living alone in the context of the Internet, compared with the elderly living with their children or with their partners, the elderly living alone are generally adapted to the use of online shopping and social software, less adapted to booking and social.

## Data availability statement

The original contributions presented in the study are included in the article/supplementary material, further inquiries can be directed to the corresponding author/s.

## Ethics statement

Ethical review and approval was not required for the study on human participants in accordance with the local legislation and institutional requirements. Written informed consent from the participants was not required to participate in this study in accordance with the national legislation and the institutional requirements.

## Author contributions

All authors listed have made a substantial, direct, and intellectual contribution to the work and approved it for publication.

## Funding

Research on the construction of a unified urban-rural basic elderly service system in Henan Province (2022BSH006), a project of Henan Province in 2022. Research on the construction path of community-based elderly care service system in Henan Province (2023-YYZD-05), a major project of philosophy and social science of Henan higher education institutions in 2022.

## Conflict of interest

The authors declare that the research was conducted in the absence of any commercial or financial relationships that could be construed as a potential conflict of interest.

## Publisher's note

All claims expressed in this article are solely those of the authors and do not necessarily represent those of their affiliated organizations, or those of the publisher, the editors and the reviewers. Any product that may be evaluated in this article, or claim that may be made by its manufacturer, is not guaranteed or endorsed by the publisher.
